# The evolution of core proteins involved in microRNA biogenesis

**DOI:** 10.1186/1471-2148-8-92

**Published:** 2008-03-25

**Authors:** Dennis Murphy, Barry Dancis, James R Brown

**Affiliations:** 1 Bioinformatics, Molecular Discovery Research, GlaxoSmithKline, 1250 South Collegeville Road, UP1345, Collegeville, Pennsylvania 19426, USA

## Abstract

**Background:**

MicroRNAs (miRNAs) are a recently discovered class of non-coding RNAs (ncRNAs) which play important roles in eukaryotic gene regulation. miRNA biogenesis and activation is a complex process involving multiple protein catalysts and involves the large macromolecular RNAi Silencing Complex or RISC. While phylogenetic analyses of miRNA genes have been previously published, the evolution of miRNA biogenesis itself has been little studied. In order to better understand the origin of miRNA processing in animals and plants, we determined the phyletic occurrences and evolutionary relationships of four major miRNA pathway protein components; Dicer, Argonaute, RISC RNA-binding proteins, and Exportin-5.

**Results:**

Phylogenetic analyses show that all four miRNA pathway proteins were derived from large multiple protein families. As an example, vertebrate and invertebrate Argonaute (Ago) proteins diverged from a larger family of PIWI/Argonaute proteins found throughout eukaryotes. Further gene duplications among vertebrates after the evolution of chordates from urochordates but prior to the emergence of fishes lead to the evolution of four Ago paralogues. Invertebrate RISC RNA-binding proteins R2D2 and Loquacious are related to other RNA-binding protein families such as Staufens as well as vertebrate-specific TAR (HIV trans-activator RNA) RNA-binding protein (TRBP) and protein kinase R-activating protein (PACT). Export of small RNAs from the nucleus, including miRNA, is facilitated by three closely related karyopherin-related nuclear transporters, Exportin-5, Exportin-1 and Exportin-T. While all three exportins have direct orthologues in deutrostomes, missing exportins in arthropods (Exportin-T) and nematodes (Exportin-5) are likely compensated by dual specificities of one of the other exportin paralogues.

**Conclusion:**

Co-opting particular isoforms from large, diverse protein families seems to be a common theme in the evolution of miRNA biogenesis. Human miRNA biogenesis proteins have direct, orthologues in cold-blooded fishes and, in some cases, urochordates and deutrostomes. However, lineage specific expansions of Dicer in plants and invertebrates as well as Argonaute and RNA-binding proteins in vertebrates suggests that novel ncRNA regulatory mechanisms can evolve in relatively short evolutionary timeframes. The occurrence of multiple homologues to RNA-binding and Argonaute/PIWI proteins also suggests the possible existence of further pathways for additional types of ncRNAs.

## Background

Recent studies have unveiled the critical roles that RNA interference (RNAi) mediated by small noncoding RNAs (ncRNAs) plays in the regulation of eukaryotic genes. One particular important ncRNA class is microRNA (miRNA), single-stranded, 19–25 nucleotide long RNAs that repress translation by binding to specific mRNA target sites. miRNAs differ from short interfering RNAs (siRNA), in that they are derived from single-stranded rather double-stranded RNA precursors. Yet like siRNAs, miRNAs can under some circumstances also effect mRNA degradation and generally share a common route to biogenesis. Computational predictions of miRNA genes and their target sites suggest that most metazoan and plant genomes encode at least several hundred if not thousands of miRNA genes and, that a large proportion of protein-coding genes have putative miRNA regulatory binding sites (reviewed in [1]).

The regulatory roles of miRNAs in both plants and animals have been reviewed in-depth elsewhere (see [2,3]). Briefly, plant miRNAs have been shown to be key regulators of tissue morphogenesis and stem development as well as mediating responses to environmental conditions [4]. In normal animal tissue, miRNA gene expression has been shown to modulate a wide variety of functions including skeletal and muscle development [5] and various metabolic pathways [6]. The abnormal expression of miRNAs has been also linked to various disease pathologies [7,8]. In cancer, miRNAs can act as either tumor suppressors or oncogenes depending upon the miRNA gene and the type of tumor [9,10]. Comparative analyses of miRNA expression profiles suggest they have potential as clinical biomarkers for the classification of tumor types [11]. Gene expression during cardiac and skeletal muscle development is also regulated by certain miRNAs which opens new opportunities for understanding muscle-related diseases [12,13]. Double stranded DNA viruses including herpes viruses, polyomaviruses and retroviruses encode their own specific miRNAs as well as interact with host miRNAs [14-16]. The differential expression of miRNAs is seen in human cells infected with viruses including HIV [17]. Their important role in disease has lead to serious consideration of miRNAs as a pharmacological target [18,19]. In agriculture, the introduction of artificial miRNAs might be a strategy for improving the resistance of crop plants to certain viruses [20].

Evolutionary analyses of miRNA gene families have revealed a combination of older ancestral relationships and recent lineage-specific diversification. The human genome itself likely encodes for a few hundred miRNAs, many of which have recognizable homologues to miRNA genes in different species (orthology) as well as amongst themselves (paralogy) [21]. Several families of miRNA genes, such as let-7, are highly conserved amongst different vertebrate and invertebrate species [22]. In addition, genomic organization of miRNA genes is often recognizable across diverse species such as the mir-196 and mir-10 gene families that likely co-evolved with Hox proteins [23] and the mir-17 gene cluster which has apparently undergone a complex series of gene duplication and loss in vertebrates [24]. However, miRNAs can also have restrictive taxonomic distribution such as the Early Embryonic microRNA Cluster (EEmiRC) locus of six pre-miRNA precursors restricted to placental (eutherian) mammals [25]. Many miRNA genes found in primates, including humans, are absent in other mammals [21,26]. Similar patterns of conservation and diversification have been observed for miRNAs in across plant species [27].

While the genomic distribution and phylogeny of miRNAs has been extensively studied, the evolution of the enabling miRNA biosynthetic pathway has received less attention. The biogenesis of a functional miRNA from its expressed gene product involves several steps and multiple proteins (for reviews see [28-31].) In animals, miRNA biogenesis begins with expression of a primary, ~1000 nt miRNA transcript, termed the pri-miRNA. From the pri-miRNA, a multi-protein complex called the Microprocessor cleaves out a ~60–70 nucleotide precursors, termed pre-miRNAs, that can fold into an imperfect stem-loop structures. There are two main components of the Microprocessor. One is called Drosha, a universal RNase III endonuclease named RNASEN in humans. The other component is a double-stranded RNA binding protein known in invertebrates as Pasha or Partner of Drosha while a similar function in vertebrates is performed by DiGeorge syndrome critical region gene 8 or DGCR8 [32]. After the pre-miRNA is cleaved from the pri-miRNA, it is transported into the cytoplasm by Exportin-5, a known transporter of RNA and protein-RNA complexes [33]. In the cytosol, the pre-miRNAs are further processed into an imperfect double stranded RNA (dsRNA) duplex by another endonuclease RNase III enzyme, Dicer [32,34].

Dicer loads mature miRNA strand into the RNA-induced silencing complex or RISC while the complementary strand, miRNA*, is degraded. Both RISC and Dicer are also known activators of siRNA. Recently, additional protein partners for Dicer and RISC have been found. The protein TRBP (human immunodeficiency virus [HIV-1] transactivating response binding protein) has been identified as a RISC partner of human Dicer [35,36] and in *Drosophila*, Loquacious, a TRBP homologue binds to Dcr1, one of two Dicer isoforms present in that species [37].

Cellular active RISC contains at least one member of the Argonaute or AGO, a large family of PIWI/PAZ domain containing proteins [38]. Structure and mutation studies suggest that in mammals, Ago2 is specifically responsible for RISC cleavage activity [39,40]. Several other proteins have also been co-purified from the RISC including RNA binding proteins VIG (Vasa Intronic Gene), Fragile X-related protein [41], nuclease Tudor-SN [42] and various helicases, like Gemin-3 and Gemin-4 [31].

While miRNA biogenesis proteins have motifs or functional domains which are conserved throughout unicellular and multicellular organisms [43], miRNA genes themselves seem to be mostly limited to metazoans and plants [44]. miRNAs have not been reported for fungi and are absent from most unicellular species including *Schizosaccharomyces pombe *and *Tetrahymena thermophila *which have known RNA silencing mechanisms [45,46]. Recently, the first occurrence of miRNAs in a unicellular organisms was reported for the single-cell algae, *Chlamydomonas reinhardtii *[47]. However, none of the *C. reinhardtii *miRNAs have any sequence homology to known plant or animal miRNAs which suggests a unique lineage-specific evolutionary occurrence – at least until further examples of miRNAs in unicellular species are found [48]. Certain double-stranded DNA viruses also have miRNAs but these were likely obtained from animal hosts via horizontal gene transfer [14]. Interestingly, the miRNA biogenesis pathway shares several proteins with the siRNA processing pathway which is found throughout both unicellular and multicellular eukaryotes [46].

Among those capable species, there are some subtle yet significant differences in miRNA function. For example, plant miRNAs are exactly complementary to their target sequence while animal miRNA are tolerant of certain base-pair mismatches [29,30]. In addition, the kinds and numbers of miRNA biogenesis proteins differ amongst various animal and plant species. Vertebrates have a single Dicer gene while the fruitfly, *Drosophila melanogaster*, has two genes, Dicer-1 (Dcr1) and Dicer-2 (Dcr2), the former of which is essential for miRNA processing [49]. Thus, there is evidence for differential evolution of miRNA biogenesis and activation pathways.

In this study, we determined the phyletic occurrences and evolutionary relationships of four main families of miRNA processing proteins: Dicer, Argonaute, double-stranded RNA-binding proteins and Exportin-5. The goal of this work was to determine whether these key miRNA proteins descended from a common early ancestor or if these genes evolved from multiple events of emergence, specialization and adaptation in specific lineages. We show that the latter scenario as the most common evolutionary theme in miRNA biogenesis. With increasing evidence that most of the vertebrate genome, including so-called junk DNA, is actively transcribed, understanding the potential for additional classes of regulatory ncRNAs is of growing importance. Thus another aim of our evolutionary analysis is to suggest the existence of other candidate ncRNA processing proteins by virtue of their relationship to known miRNA pathway proteins.

## Results and Discussion

### Dicer Evolution

RNaseIII enzymes are categorized into three classes, all of which contain at least one catalytic domain. Class I, found in bacteria and yeast, is the simplest having only a single RNaseIII domain and a double-stranded RNA (dsRNA) binding domain. Class II and Class III enzymes commonly have a second RNaseIII domain but are distinguishable from each other by specific auxiliary N-terminal domains. Drosha, a Class II enzyme, has proline-rich and arginine-serine (RS) domains while Dicer, a Class III enzyme, has helicase and PAZ (Piwi/Argonaute/Zwille) domains. As the name indicates, the PAZ domain is also found in Argonaute proteins, another essential group of RNAi processing enzymes.

**Figure 1:**
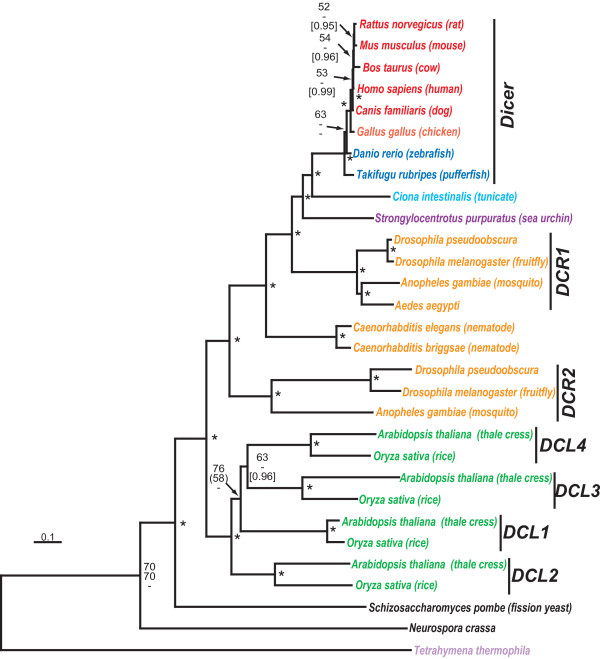
Neighbor-joining phylogenetic tree of the Dicer protein family. Major organism groups (with colours) are mammals (red), birds (light red), cold-blooded vertebrates (deep blue), urochordates (light blue), deutrostome invertebrates (purple), protostome invertebrates (orange). plants (green), fungi (black), and protists (light purple). Stacks of three numbers show, in descending order, the percent occurrence of nodes in greater than 50% of 1000 bootstrap replicates of neighbor joining (plain text) and maximum parsimony (italicized text) or Bayesian posterior probability (only 0.90 or greater, in square parentheses). Asterisks ("*") indicate those nodes supported 60% or greater by the first two tree-building methods and 0.95 Bayesian posterior probability. Nodes with one or two values less than 50% have dashes ("-") while values less than 50% are unmarked. Scale bar represents 0.1 expected amino acid residue substitutions per site. The multiple sequence alignment file is given in Additional file 1.

Class III or Dicer-like RNaseIII enzymes are found throughout eukaryotes (Fig. 1) although the number of Dicer homologues is variable among different groups [see Additional file 1]. Protists such as the ciliate, *Tetrahymena thermophila*, and fungi have a single copy of Dicer [50]. Plants have four Dicer homologues, called DCL1–4, each one specialized for handling a specific small RNA [51] with DCL1 responsible for processing mature miRNAs from their primary transcripts [30]. The remaining plant Dicers could function in anti-viral defense [52]. Our phylogenetic tree suggests the scenario of early gene duplication in plants because all four Dicers isoforms are found in the genomes of both rice (*Oryza sativa*) and thale crest (*Arabidopsis thaliana*) and show orthologous relationships.

In animals, the evolutionary situation is a little more complicated. Single Dicer genes occur in mammals and cold-blooded vertebrates which have direct orthologues in urochordates (represented in Fig. 1 by the tunicate, *Ciona intestinalis*) and deutrostomes (represented by the sea urchin, *Strongylocentrotus purpuratus*). Among protostomes, nematodes (*Caenorhabditis *sp.) have a single Dicer gene while *Drosophila *species, mosquito (*Anopheles gambiae *and *Aedes aegypti*) and possibly all arthropods, have two Dicer genes, *Dcr1 *and *Dcr2*. Unlike the situation for plants, insect Dicer gene duplications do not correspond with the divergence of arthropods from other metazoans since nematode Dicer splits the insect clade in the phylogenetic tree with significant bootstrap support (Fig. 1). Rather, DCR2 seems to be a more divergent group of RNaseIII enzymes which, in our tree, is basal to all other metazoan Dicer proteins. *Drosophila *and other insects have three RNaseIII enzymes, DCR1, DCR2 and Drosha. Mutational studies have shown that the *Dcr1 *gene is essential for miRNA in fruitfly [49], but not dsRNA processing. The converse is true for the *Dcr2 *gene where mutants have normal miRNA levels but have abnormal processing of dsRNAs. *Drosophila *Dicer is just one example of lineage-specific gene duplication and specialization in miRNA biogenesis over the course of eukaryotic evolution.

### Argonaute Evolution

At the core of the RISC is Argonaute (AGO), highly basic ~100 kD proteins characterized by PAZ and PIWI domains. The N-terminal PAZ domain, also found in Dicer, is about 130 amino acids in length and is thought to function in protein-protein interactions (see review [38]). The C-terminal PIWI domain is approximately 300 amino acids in length. The exact functioning of these domains in miRNA processing is unknown although some clues have been revealed in recent structures of a PIWI-domain protein (AfPiwi) from the thermophilic Archaea, *Archaeoglobus fulgidus*, in complex with a small siRNA-like duplex [53,54]. The miRNA seed region, comprised of nucleotides 2–8, is critical for target recognition [55]. In the AfPiwi structure, the first nucleotide of the siRNA-like substrate is also unbound to the target sequence and locked into the protein binding pocket. The AfPiwi is an imperfect model for eukaryotic AGO because it lacks a PAZ domain. However, other evidence suggests that PAZ domains bind to 3' OH terminal ends of RNA or duplexes with 3' overhang [56]. Therefore, PAZ and PIWI likely serve to align and stabilize small RNAs to their respective mRNA target sequences. The stabilized duplex with mRNA is subsequently either cleaved by siRNA or translationally repressed by miRNA.

**Figure 2:**
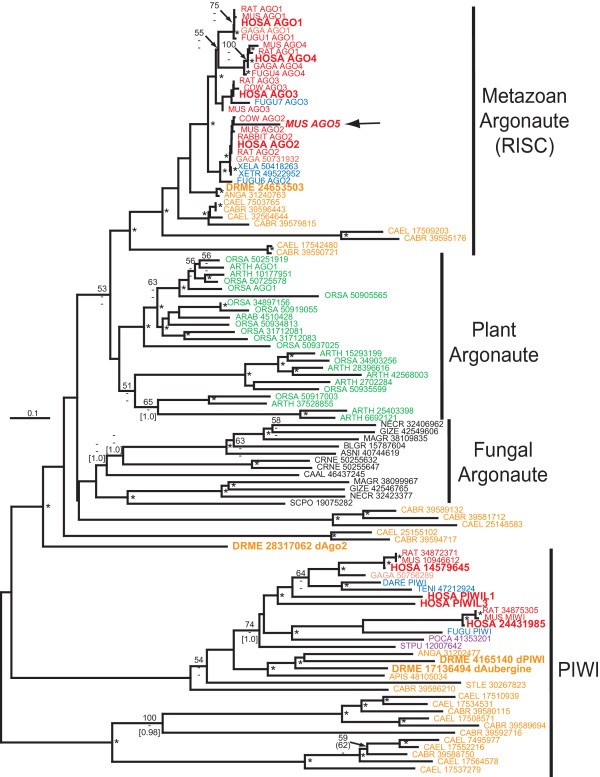
Neighbor-joining phylogenetic tree of Argonaute/PIWI protein family. Major protein subgroups are labeled. The tree is unrooted. Phylogenetic reconstruction method, species colour-coding and nodes labeling of significance are the same as Fig. 1. Human and Drososphila PIWI/Ago proteins discussed in the text are in larger font. The branch leading to a putative, but unlikely, fifth Argonaute gene homolog in mouse, mAgo5, is labeled with a large arrow (see text for explanation). Other branches are labeled by a four letter species identifier (the first two letters from the genus and species names) and the GenBank accession number). Species name abbreviations are given in the Methods. The multiple sequence alignment file is given in Additional file 2.

The AGO family is highly diverse with multiple, identifiable variants in plants, fungi, invertebrates and vertebrates. Phylogenetic analysis shows two distinct groups comprised of Argonaute and PIWI type proteins. Within the Argonaute cluster, the RISC-associated Argonaute proteins of metazoans are monophyletic relative to other AGO members (Fig. 2). Plant and fungal Argonaute-like AGO proteins seem to form separate groups although bootstrap values are low supporting these clades are low. For the PIWI cluster, evolutionary analysis also suggests that the multiple PIWI proteins in mammals, including humans, arose from vertebrate-specific gene duplication events. Multiple PIWI-like proteins in nematodes evolved from various lineage-specific gene duplications events separate from other animals. [see Additional file 2].

**Figure 3:**
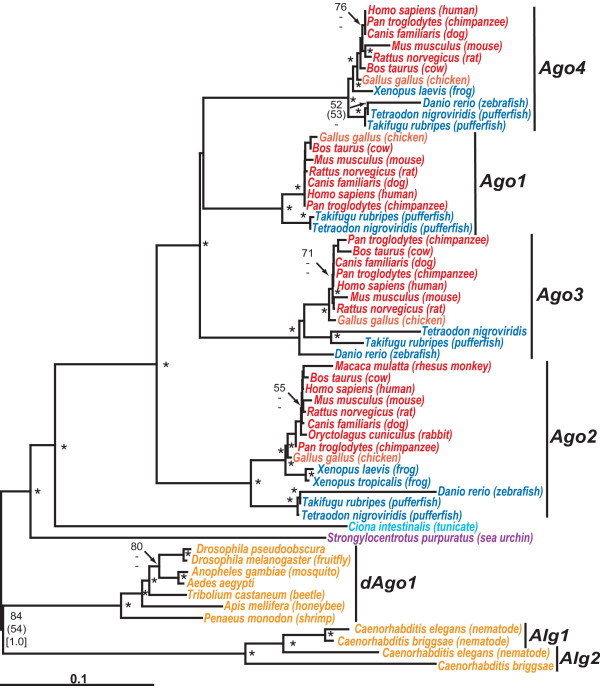
Phylogenetic tree of vertebrate Argonaute rooted by closely related invertebrate homologues. The major vertebrate subgroups are: Argonaute 1/eukaryotic translation initiation factor 2C, 1 (Eif2c1) [Ago1], Argonaute 2/EIF2C2 [Ago2], Argonaute 3/EIF2C3 [Ago3] and Argonaute 4/EIF2C2 [Ago4]. Phylogenetic reconstruction method, species colour-coding and nodes labeling of significance are the same as Fig. 1. The multiple sequence alignment file is given in Additional file 3.

More detailed phylogenetic analysis (Fig. 3) show that humans and other vertebrates have four Argonaute genes called *Ago1–4*, also known as *eIFC1–4 *for their putative regulatory role in translation [see Additional file 3]. Included in this subfamily is human AGO1/EIF2C1, once called GERp95 because of its sub-cellular localization in the endoplasmic reticulum or ER [57]. In the RISC, AGO2 catalyzes RNA cleavage targeted by siRNAs and miRNAs [39]. AGO1 and AGO2 appear to be cellular localized to specific mRNA decay centers that are known as cytoplasmic bodies [58]. The roles of AGO3 and AGO4 are still unclear although they might support aspects of cell differentiation in multi-cellular organisms such as neural development [59].

In *Drosophila*, four AGO-like proteins have been identified which are dPiwi, dAubergine, dAGO1 and dAGO2 [38]. dPiwi and dAubergine are expressed in embryos and appear to affect germline development. Phylogenetic analysis places dPiwi and dAubergine with other arthropod PIWI proteins which is the outgroup to vertebrate PIWI/MIWI proteins (Fig. 2). Both dAGO1 and dAGO2 are RISC components but with different small RNA specificities. Okamura *et al*.[60] showed that Drosophila embryos lacking dAGO2 were siRNA-directed RNAi-defective but still capable of miRNA-directed target RNA cleavage. In contrast, dAGO1 deficient mutants were incapable of producing mature miRNAs while siRNA-directed target RNA cleavage was intact. Consistent with their findings, our phylogenetic analysis shows that of the four Drosophila Argonaute homologues, dAGO1 is most closely related to RISC associated AGO proteins involved with miRNA processing in vertebrates while dAGO2 is highly divergent. Nematodes also have multiple PIWI/Argonaute proteins of which two, Alg1 and Alg2, are the immediate outgroup to insect and vertebrate AGO proteins (Fig. 2 &3). The different roles of these proteins is unknown although indirect evidence suggests Alg1 might be recruited into the miRNA RISC [61].

Humans have eight Argonaute-like proteins [62], four of which fall into the wider PIWI family while the remainder are AGO proteins with orthologues in other mammals and vertebrates (Fig. 2). Homologues in insects and nematodes are clearly outgroups to all four vertebrate AGO isoforms (Fig. 3). All four mammalian genes AGO1–4 (EIF2C1–4) have orthologues in cold-blooded vertebrates (i.e. fish and amphibians). The urochordates (*C. intestinalis*) and deutrostomes (*S. purpuratus*), have only single AGO copies which appear ancestral to all vertebrate AGOs. In summary, phylogenetic analysis suggests that there was an early chordate radiation of the Argonaute gene family, possibly with the miRNA component AGO2 as the ancestor to the other three AGO proteins. In humans, AGO1, AGO3 and AGO4 are closely clustered together on chromosome 1 which also suggests their common evolution from a series of concurrent gene duplications.

The existence of other Argonaute/PIWI proteins leads to speculation that additional RISC-like ncRNA processing complexes might be found. Indeed, two recent reports describe a novel class of small RNAs isolated from mouse testis libraries which bind to two PIWI proteins, MIWI [63] and MILI [64] (see Fig 2 for the tentative phylogenetic position of vertebrate PIWI/MIWI proteins). These "PIWI-interacting RNAs" called piRNAs, might number in the thousands and appear to be encoded by specific genomic regions that are also conserved in rat and human. The biochemical processing of piRNAs as well as their putative regulatory functions are presently not well understood.

It should be noted that a fifth mammalian Argonaute gene called mAgo5 (GenBank accession no. AAN75582) has been reported for the mouse. This protein was identified through a homology search of the initial mouse genomic sequence made available by subscription from the company, Celera [38]. The mAgo5 open reading frame (ORF) is fragmented with some regions being either highly divergent or deleted relative to other Argonaute proteins. Our phylogenetic analysis of the entire Argonaute/PIWI protein positions mAgo5 as a particularly long branch within the AGO2 cluster, close to the confirmed mouse AGO2 protein (Fig. 2). However, in a phylogenetic analysis restricted to metazoan Argonaute proteins, mAgo5 was the most divergent sequence and landed as the outgroup to both vertebrates and invertebrates (not shown) which suggests that its position in the full Argonauete/PIWI tree is an artifact. Our sequence database searches failed to reveal any mAgo5 orthologue in other mammals or cold-blooded vertebrates. Therefore, we suggest that unless confirmed by re-sequencing of genomic DNA, mAgo5 is likely an artifact from homology searches of incomplete DNA sequence assemblies of the Celera mouse genome.

### Evolution of TRBP, Loquacious and Other RNA-binding Proteins

**Figure 4:**
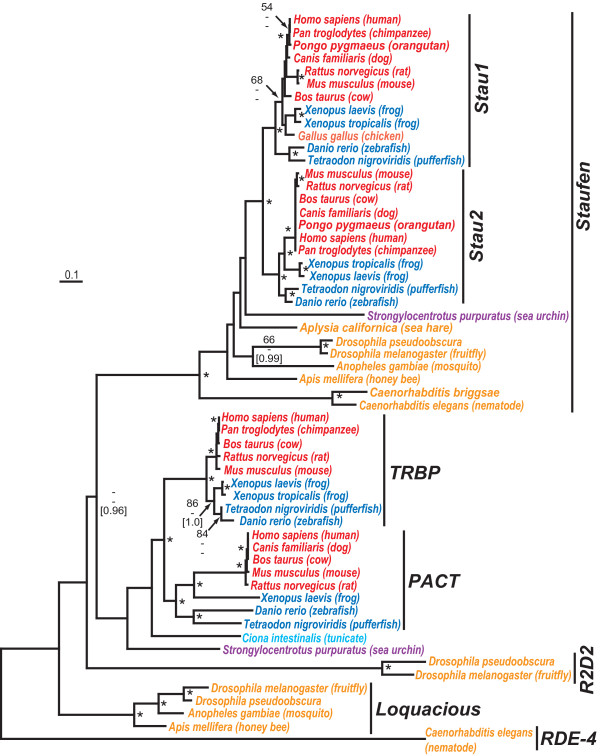
Neighbor-joining phylogenetic tree of double-stranded RNA-binding proteins. Major clusters of proteins include Staufen subfamilies (Stau1 and Stau2), HIV trans-activator RNA (TAR), RNA-binding protein (TRBP), protein kinase R (PKR)-activating protein (PACT), Loquacious and R2D2. Phylogenetic reconstruction method, species colour-coding and nodes labeling of significance are the same as Fig. 1. The multiple sequence alignment file is given in Additional file 4.

Mammalian TAR (HIV trans-activator RNA) RNA-binding protein or TRBP is essential for the recruitment of Dicer-complexed miRNAs to RISC AGO2 [35,36]. In *Drosophila*, a homologous protein to TRBP called Loquacious binds to DCR-1 to facilitate the normal processing of pre-miRNAs [37]. Both TRBP and Loquacious, with three dsRNA-binding domains, are distantly related to Drosophila R2D2, another dsRNA binding protein shown to heterodimerize with DCR-2 [65] [see Additional file 4]. In *C. elegans*, RDE-4 is a comparable dsRNA binding protein that interacts with DCR-1 and is essential for RNAi processing [66]. In vertebrates, TRBP is a paralogue to the protein kinase R (PKR)-activating protein or PACT [36,67] (Fig. 4). Both proteins regulate PKR, a dsRNA-regulated interferon-inducible protein kinase but with counteracting effects – TRBP is an inhibitor of PKR while PACT is an activator [68]. TRBP is also involved in HIV-1 gene expression [69] which raises intriguing possibilities about the linkage between miRNAs and the response of the IFN-PKR pathway to HIV-1 infection [67].

Our phylogenetic tree shows that *Drosophila *Loquacious, also found in other insects, is ancestral to both TRBP and PACT. Sea urchin and tunicate have single genes which appear to be evolutionary intermediates between invertebrate Loquacious and vertebrate TRBP/PACT. However, bootstrap and posterior probability support for this branching order is low which might reflect either the available partial amino acid sequences (at the time of manuscript submission, both sea urchin and tunicate genomes were incomplete) or the need for more extensive taxonomic sampling. Regardless, TRBP and PACT genes likely diverged in very early chordates since cold-blooded vertebrates, the fishes and amphibians, as well as mammalians have full complements of these genes. As suggested by its evolutionary relationships, PACT has been recently implicated in small RNA processing in partnership with TRBP and dicer [70].

Sequence database searches using TRBP revealed other related dsRNA binding proteins. As mentioned above, Drosophila R2D2 and *C. elegans *RDE-4, both known participants in RNAi processing, are distantly related to Loquacious, TRBP and PACT. Other evolutionary related dsRNA binding proteins are the Staufens, a family of proteins with a tubulin-binding domain which likely serve to transport mRNAs intra-cellularly using microtubules. There are two families of Staufens in mammals, Stau1 and Stau2, which seem to have specific functions in mRNA transport in neurons [71]. In *Drosophila*, Staufens have been associated with a number of neurological functions including neurodegeneration [72] and long-term memory formation [73]. Like miRNA precursors, Staufen-dsRNA complexes are transported out of the nucleus by Exportin-5 [74]. Although Staufens have not been previously linked with ncRNAs, their similarity to three known dsRNA binding protein families in the miRNA pathway suggests that further study about their potential role in small RNA transport might be warranted. Recent studies suggest that Staufen-containing neuronal granules share several protein components, such as Me31B, with cytoplasmic P-bodies which are thought to be the sites for translational regulation by miRNA [75,76].

### Evolution of Exportin-5

The transfer of RNAs and proteins between the nucleus and the cytoplasm is facilitated by shuttling transporters which have specificity for various cargoes (reviewed in [77-79]). The importin-β family is a large group of karyopherin-related nuclear transporters, which includes proteins that facilitate both nuclear import (importins) and nuclear export (exportins). The directionality of transport in the importin-β family is determined by interactions with the small nuclear GTPase, Ran.

**Figure 5:**
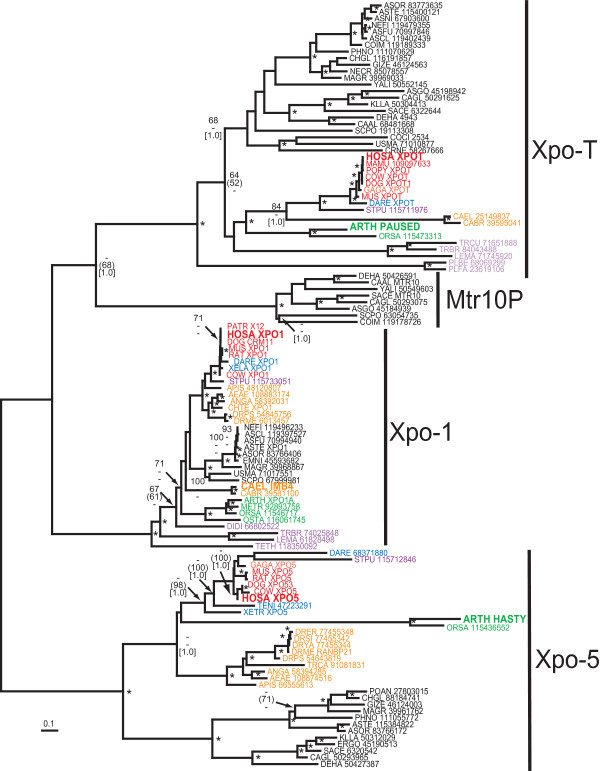
Neighbor-joining phylogenetic tree of exportins and importins that are most closely related to Exportin-5. The families are Exportin-5 (Xpo-5), Exportin-1 (Xpo-1), Exportin T (Xpo-T), and fungal importin Mtr10P. Phylogenetic reconstruction method, species colour-coding and nodes labeling of significance are the same as Fig. 1. The tree is unrooted. Locations of some specific isoforms from *Homo sapiens*, *Arabidopsis thaliana *and *Caenorhabditis elegans *(CE) that are mentioned in the text are annotated on the tree. Species name abbreviations are given in the Methods. The multiple sequence alignment file is given in Additional file 5.

In most species, Exportin-5 is responsible for shuttling pre-miRNA out of the nucleus into the cytoplasm. Exportin-5 also transports other small RNAs and several protein binding partners have been identified including ILF3 (Interleukin enhancer binding factor 3) [80], the ILF3-binding protein JAZ [81], and the previously mentioned RNA-binding protein Staufen2 [74]. Homology searches and phylogenetic analysis revealed three closely related paralogous yet monophyletic separate families to Exportin-5 which are Exportin-1 and Exportin-T, transporters specific for snRNAs and tRNAs, respectively, and Mtr10P, fungi-specific nuclear importins [77] (Fig. 5; see Additional file 5).

Aside from fungal Mtr10P, the three remaining exportins have an unusual phyletic occurrence. Exportin-1, Exportin-T and Exportin-5, are all encoded by the genomes of plants, cold-blooded vertebrates and mammals as well as fungi. Partial sequences corresponding to all three exportins were also found in the urochordate, *Ciona intestinalis*, although the lengths of contiguous sequences were too short for phylogenetic reconstruction (data not shown). Moreover, orthologues of all three exportins were identified in the sea urchin suggesting commonality across deutrostomes. In *Arabidopsis*, the Exportin-5 protein Hasty has been shown to transport miRNA [82] while the Exportin-T-like transporter, Paused facilitates tRNA export from the nucleus [83].

However, among protostome invertebrates there are some notable examples of missing exportins and shifts in RNA specificity. Drosophila exportin-5 also transports tRNAs which might compensate for the lack of exportin-T across arthropods [84]. The nematodes, *C. elegans *and *C. briggsae*, lack Exportin-5 but have orthologues to Exportin-T and Exportin-1, the latter also called IMB-4 (Fig. 5). It is presently unclear how nematodes actually export miRNAs from the nucleus without Exportin-5, but IMB-4 or Exportin-T are possible candidates for this function [85].

**Figure 6:**
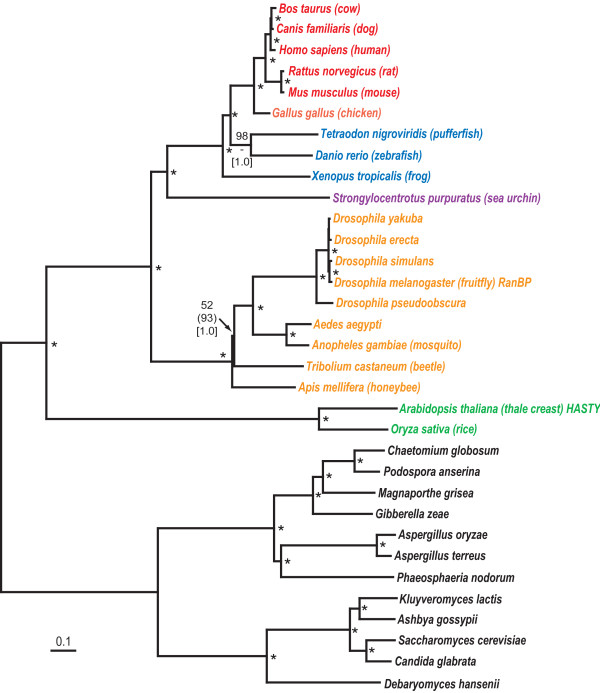
Neighbor-joining phylogenetic tree of Exportin-5 orthologues. Phylogenetic reconstruction method, species colour-coding and nodes labeling of significance are the same as Fig. 1. The multiple sequence alignment file is given in Additional file 6.

Based on current genome sequences, fungi, plants and deutrostomal metazoans have all three exportins while nematodes and arthropods (possibly all protostomes) lack full complements. Subtree analysis of Exportin-5 showed generally expected pattern of species evolutionary relationships with deutrostomes, protostomes, plants and fungi forming separate monophyletic groups (Fig. 6; see Additional file 6). Exportin-T and Exportin-1 also show clustering by taxonomic group (Fig. 5). The most parsimonious explanation for this unusual phyletic distribution of exportin genes is the independent loss and shifting of function between paralogues in the early evolution of certain invertebrate groups. In arthropods, the loss of Exportin-T was compensated by Exportin-5 adapting a dual specificity transport role for tRNAs as well as miRNAs. In nematodes, either IMB-4 or Exportin-T possibly fulfills the role of miRNA transport in the absence of Exportin-5.

Not surprisingly, putative Exportin-T and Exportin-1 homologues which function to shuttle other small RNAs besides miRNAs were also found in protists, such as species of Plasmodium, Trypanosomes and Leishmania. Other components of the RNA-silencing pathway have been detected in these species. However, no plausible miRNA gene orthologues have been detected outside of the metazoan [45] except for the unicellular algae, *Chlamydomonas reinhardtii *[47] Interestingly, our database search shows that fungi, which have regulation by siRNA but not miRNA, have genes encoding for Exportin-5, Exportin-1, and Exportin-T.

### Discussion – Something Borrowed; Something New

There are many additional proteins involved in miRNA biogenesis and a thorough evolutionary analysis of all is beyond the scope of this report. However, preliminary phylogenetic trees for several other components (data not shown) show similarly diverse evolutionary patterns as Argonaute, Dicer, dsRNA-binding proteins and Exportin-5. Gemin4, Gemin5, and Tudor-SN are other examples where particular members of multi-protein families have specific roles in miRNA and siRNA processing and activation. In contrast, *Drosophila *Pasha and vertebrate DGCR8 are direct orthologues, without any lineage-specific gene duplications.

**Figure 7:**
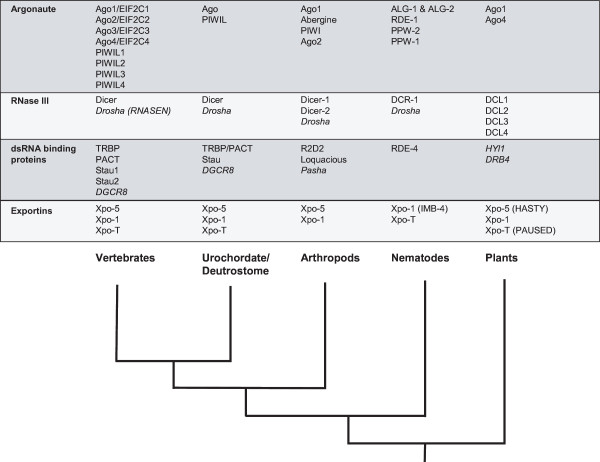
Phyletic distribution of microRNA biogenesis proteins from this study. Representative species for the taxonomic groups are *Homo sapiens *(vertebrates), *Ciona intestinalis *(urochordates), *Strongylocentrotus purpuratus *(echinoderms), *Drosophila melanogaster *(arthropods), *Caenorhabditis elegans *(nematodes), and, *Arabidopsis thaliana *(plants). Cladogram at the bottom represents relative evolutionary relationships among these groups according to the Tree of Life web project [96]. In italics are a few proteins (Drosha, DGCR8, Pasha HYI1 and DRB4) which were not included in the phylogenetic analyses but are known miRNA or siRNA processing enzymes [2,3].

Co-opting particular isoforms from large, diverse protein families seems to be a common theme in miRNA biogenesis (Fig. 7). Arthropods have two Dicer isoforms with distinct roles; DCR-1 is functional in miRNA processing while DCR-2 is essential for siRNA activation [49]. In plants, there are four Dicer paralogues which have specialized functions involving different types of host and viral RNAs [51,52]. Similarly, of the four vertebrate AGO proteins, AGO2 alone is essential for RISC catalytic activity. The occurrence of similar yet divergent miRNA biogenesis proteins in vertebrates, invertebrates, and plants suggests that translational regulation by miRNAs has undergone significant lineage-specific modifications. A thorough knowledge of these underlying evolutionary patterns might be an important caveat when comparing miRNA-related experiments from different model systems. More generally, the variable recruitment and adaptations of proteins for enabling miRNA biogenesis across species further reveals the extensive plasticity of genomes for rapidly evolving novel yet significant cellular regulatory networks.

Different natural selection pressures between and within species might have also played a role in the occurrence of miRNA pathway genes and their levels of sequence divergence. Obbard et al. [86] recently showed that the siRNA pathway genes *Dcr2*, *R2D2 *and *Ago2 *are evolving rapidly in *Drosophila *which they provocatively suggest might a consequence of an antiviral "arms-race". Conversely, they demonstrate that the rates of amino acid substitutions were not elevated in miRNA associated genes, *Ago2 *and *Dcr1 *which might be due to constraints associated with the essential regulatory roles of miRNAs in many cellular functions.

The components of siRNA processing are ancient as evident from proteins with distant but recognizable motifs found in single-cell eukaryotes and, the case of Dicer, even bacteria and archaea have similar RNaseIII domains [46]. However, genes encoding proteins specific for miRNA biogenesis seem to be more recent innovations. The diversity of miRNA pathway genes in plants, as well as metazoan protostomes, and deutrostomes, suggests that these three lineages had specific adaptations (Fig. 7). Two reports [45,87] noted that further expansions of miRNA gene families themselves might have coincided with the emergence of bilaterians, vertebrates and mammals.

Our comparative genomic analyses show that urochordates (represented by the tunicate, *C. intestinalis*) and early deutrostomes (represented by the purple sea urchin, *S. purpuratus*) have single copies of vertebrate-like miRNA processing proteins. Provisional that no further gene copies are found once their entire genomes are completely sequenced, these species may represent the ancestral state of the deutrostome/chordate miRNA pathway which subsequently underwent specialization *via *gene duplications in early vertebrates. According to robust Bayesian phylogenetic analysis of multiple proteins concurrently calibrated with the fossil record, echinoderms (sea urchins and seastars) and protochordates (cephalochordates) diverged about 896 million years ago (MYA)[88]. Chordates and urochordates split more recently, about 794 MYA. In the most recent release of miRBASE (Sanger Center, release 10.1), there are no miRNAs reported for either urochordates or echinoderms. However, Hertel et al. [45] identified by homology searches, 40 new miRNA genes in *S. purpuratus *and 9 miRNA genes in two species of tunicates *C. intestinalis *and *C. savignyii*. Collectively with our analyses showing that miRNA biogenesis proteins are found in tunicates and sea urchins, these species probably do have rudimentary miRNA regulatory networks. The potential simplicity yet close vertebrate similarity of these species suggests that they might be intriguing systems to study the structure, function and evolution of miRNAs. Moreover, as genomic data is generated for intermediate groups between urochordates and jawed-fishes such as the jawless fishes (i.e. hagfish and lampreys), we might gain more insight into the specific stages in the evolution of the vertebrate miRNA pathway.

## Conclusion

Finally, our study suggests that there are several other candidate proteins for processing small, ncRNAs. Indeed, divergent homologues to miRNA processing AGO2, the PIWI proteins, MIWI and MILI, have been recently shown to process a novel class of ncRNAs, the "PIWI-interacting RNAs" or piRNAs. Our study suggests that there are multiple Argonaute/PIWI as well as double-stranded RNA-binding proteins and exportins which, by evolutionary associations, are hypothesized to participate in the processing of additional classes of ncRNAs and might warrant further experimental investigation.

## Methods

Protein (amino acid) sequences were retrieved from GenBank Nonredundant and species-specific databases (*Ciona intestinalis *[tunicate – urochordate] and *Strongylocentrotus purpuratus *[sea urchin – early deutrostome]) via BLASTP (default settings) searches using human miRNA pathway genes as the initial queries [89]. As necessary, sequences from other species or additional paralogues (i.e. such as Exportin-T) were used to obtain a full set of homologues. Homology cut-offs were E-values ≤ 10e-10.

Initial multiple sequence alignments were performed using the program CLUSTALW v1.7 [90] with default settings and subsequently, refined manually using the program SEQLAB of the GCG Wisconsin Package v11.0 software package (Accelrys, San Diego, CA, USA). We removed regions with residues that could not be unambiguously aligned or that contained insertions or deletions. Multiple sequence alignments are included as Additional files 1, 2, 3, 4, 5, 6. For each file, the first row titled "Analysis_1", marks with an "*" the columns of amino acids retained in the edited multiple sequence alignments for phylogenetic analysis.

We constructed phylogenetic trees using distance neighbor-joining (NJ), maximum parsimony (MP), and Bayesian posterior probabilities (BP). NJ trees were based on pair wise distances between amino acid sequences using the programs NEIGHBOR and PROTDIST (Dayhoff option) of the PHYLIP 3.6 package [91]. The programs SEQBOOT and CONSENSE were used to estimate the confidence limits of branching points from 1000 bootstrap replications. MP analysis was performed using PAUP4.0b5 software [92] where the number and lengths of minimal trees were estimated from 100 random sequence additions, while confidence limits of branch points were estimated by 1000 bootstrap replications. BP trees were constructed using the software MrBayes v3.0B4 [93,94]. Bayesian analysis used the mixed model of sequence evolution with random starting trees. Markov chains were run for 10^6 ^generations, burn-in values were set for 10^4 ^generations, and trees sampled every 100 generations. All trees were visualized using the program TREEVIEW v1.6.6 [95]. Subsets of Argonaute and Exportin protein family members that were known to be involved in miRNA biogenesis were also re-aligned and subjected to separate phylogenetic analysis.

The Dicer phylogeny shown in Fig. 1 was based on an edited alignment of 926 amino acids. One minimal length MP trees were recovered, 3020 steps in length with a consistency index (CI) of 0.6907 and a retention index (RI) of 0.6629. The Argonaute/PIWI phylogeny shown in Fig. 2 was based on an edited alignment of 288 amino acids. MP analysis recovered 214 minimal length trees, 6035 steps in length with a consistency index (CI) of 0.3934 and a retention index (RI) of 0.6699. The variable branch arrangements were among certain terminal nodes within of the each AGO protein.

The animal Argonaute phylogeny shown in Fig. 3 was based on an edited alignment of 834 amino acids. MP analysis recovered 200 minimal length trees, 2538 steps in length with a consistency index (CI) of 0.7281 and a retention index (RI) of 0.8938. The variable branch arrangements were among certain terminal nodes (mammals) within of the each vertebrate Ago families, Ago1–4. However, separate monophyly of each Ago family was strongly supported by all phylogenetic methods. The double-stranded RNA-binding protein tree shown in Fig. 4 was based on an edited alignment of 156 amino acids. MP analysis recovered 34 minimal length trees, 1334 steps in length with a consistency index (CI) of 0.6402 and a retention index (RI) of 0.8294. The variable branch arrangements were among certain terminal nodes within vertebrate clades of Staufens, PACT and TRBP which did not affect the central findings. The Exportin/Mtr10P phylogeny shown in Fig. 5 was based on an edited alignment of 392 amino acids. MP analysis recovered 20025 minimal length trees, 10368 steps in length with a consistency index (CI) of 0.4169 and a retention index (RI) of 0.7567. The variable branching concerned terminal branches which did not affect the main observations of this tree. The Exportin 5 phylogeny Fig. 6 was based on an edited alignment of 809 amino acids. MP analysis recovered four minimal length trees, 6186 steps in length with a consistency index (CI) of 0.7118 and a retention index (RI) of 0.8110. The variable branching concerned the two fish Exportin 5 proteins relative to each other which, also, did not affect the central findings.

For Fig. 2, the species (with abbreviations) included in the tree are *Anopheles gambiae *(ANGA), *Apis mellifera *(APIS), *Arabidopsis thaliana *(ARTH), *Aspergillus nidulans *(ASNI), *Blumeria graminis *(BLGR), *Candida albicans *(CAAL), *Bos taurus *(COW), *Cryptococcus neoformans *(CRNE), *Danio rerio *(DARE), *Drosophila melanogaster *(DRME), *Takifugu ribripes *(FUGU), *Gallus gallus *(GAGA), *Gibberella zeae *(GIZE), *Homo sapiens *(HOSA), *Magnaporthe grisea *(MAGR), *Mus musculus *(MUS), *Neurospora crassa *(NECR), *Oryza sativa *(ORSA), *Podocoryne carnea *(POCA), *Oryctolagus cuniculus *(RABBIT), *Rattus norvegicus *(RAT), *Schizosaccharomyces pombe *(SCPO), *Stylonychia lemnae *(STLE), *Strongylocentrotus purpuratus *(STPU), *Tetraodon nigroviridis *(TENI), *Xenopus laevis *(XELA), and *Xenopus tropicalis *(XETR).

For Fig. 5, species (and abbreviations) included in the tree are *Aedes aegypti *(AEAE), *Anopheles gambiae *(ANGA), *Apis mellifera *(APIS), *Arabidopsis thaliana *(ARTH), *Aspergillus clavatus *(ASCL), *Aspergillus fumigatus *(ASFU), *Ashbya gossypii *(ASGO), *Aspergillus nidulans *(ASNI), *Aspergillus oryzae *(ASOR), *Aspergillus terreus *(ASTE), *Candida albicans *(CAAL), *Candida glabrata *(CAGL), *Chaetomium globosum *(CHGL), *Chironomus tentans *(CHTE), *Bos taurus *(COW), *Coprinopsis cinerea *(COCI), *Coccidioides immitis *(COIM), *Cryptococcus neoformans *(CRNE), *Danio rerio *(DARE), *Debaryomyces hansenii *(DEHA), *Dictyostelium discoideum *(DIDI), *Canis lupus familiaris *(DOG), *Drosophila erecta *(DRER), *Drosophila melanogaster *(DRME), *Drosophila pseudoobscura *(DRPS), *Drosophila simulans *(DRSI), *Drosophila yakuba *(DRYA), *Emericella nidulans *(EMNI), *Ashbya gossypii *[*Eremothecium gossypii*] (ERGO), *Gallus gallus *(GAGA), *Gibberella zeae *(GIZE), *Homo sapiens *(HOSA), *Kluyveromyces lactis *(KLLA), *Leishmania major *(LEMA), *Macaca mulatta *(MAMU), *Magnaporthe grisea *(MAGR), *Medicago truncatula *(METR), *Mus musculus *(MUS), *Neosartorya fischeri *(NEFI), *Neurospora crassa *(NECR), *Oryza sativa *(ORSA), *Ostreococcus tauri *(OSTA), *Pan troglodytes *(PATR), *Phaeosphaeria nodorum *(PHNP), *Plasmodium berghei *(PLBE), *Plasmodium falciparum *(PLFA), *Podospora anserine *(POAN), *Pongo pygmaeus *(POPY), *Rattus norvegicus *(RAT), *Saccharomyces cerevisiae *(SACE), *Schizosaccharomyces pombe *(SCPO), *Strongylocentrotus purpuratus *(STPU), *Tetraodon nigroviridis *(TENI), *Tetrahymena thermophila *(TETH), *Trypanosoma brucei *(TRBU), *Tribolium castaneum *(TRCA), *Trypanosoma cruzi *(TRCU), *Ustilago maydis *(USMA), *Xenopus laevis *(XELA), *Xenopus tropicalis *(XETR), and *Yarrowia lipolytica *(YALI).

## Competing interests

The author(s) declare that they have no competing interests.

## Authors' contributions

JRB conceived the study, performed some of the phylogenetic analysis and drafted the manuscript. DM compiled several gene families, performed additional phylogenetic analysis and contributed to the manuscript. BD performed additional database searches and phylogenetic analysis. All authors have read and approved the final manuscript.

## Supplementary Material

Additional file 1 Dicer protein sequences. Multiple sequence alignment of Dicer protein sequences used for phylogenetic tree reconstruction. For each file, the first row titled "Analysis_1", marks with an "*" the columns of amino acids retained in the edited multiple sequence alignments for phylogenetic analysis. Click here for file

Additional file 2 Argonaute/PIWI protein sequences. Multiple sequence alignment of PIWI and Argonaute protein sequences used for phylogenetic tree reconstruction. See Fig. 2 caption for the definitions of species abbreviations. Click here for file

Additional file 3 Argonaute protein sequences. Multiple sequence alignment of Argonaute protein sequences used for phylogenetic tree reconstruction. Click here for file

Additional file 4 Double-stranded RNA-binding proteins. Multiple sequence alignment of Double-stranded RNA-binding protein sequences used for phylogenetic tree reconstruction. Click here for file

Additional file 5 Exportin family proteins. Multiple sequence alignment of Exportin-5, Exportin-1 and Exportin-T protein sequences used for phylogenetic tree reconstruction. See Fig. 5 caption for the definitions of species abbreviations. Click here for file

Additional file 6 Exportin-5 proteins. Multiple sequence alignment of Exportin-5 protein sequences used for phylogenetic tree reconstruction. Click here for file
